# Incidence of premature battery depletion in subcutaneous cardioverter-defibrillator patients: insights from a multicenter registry

**DOI:** 10.1007/s10840-023-01468-1

**Published:** 2023-01-18

**Authors:** Jakob Lüker, Marc Strik, Jason G. Andrade, Alexandre Raymond-Paquin, Mohamed Hassan Elrefai, Paul R. Roberts, Óscar Cano Pérez, Jordana Kron, Jayanthi Koneru, Hilton Franqui-Rivera, Arian Sultan, Angela Ernst, Jörn Schmitt, Alexander Pott, Christian Veltmann, Neil T Srinivasan, Jason Collinson, Antonius M. W. van Stipdonk, Dominik Linz, Nina Fluschnik, Tobias Tönnis, Andreas Haeberlin, Sylvain Ploux, Daniel Steven

**Affiliations:** 1 Department of Electrophysiology, University of Cologne, University Hospital Cologne, Kerpener Strasse 62, 50937 Cologne, Germany; 2 Cardio-Thoracic Unit, Bordeaux University Hospital (CHU), Pessac-, Bordeaux, France; 3 IHU Liryc, Electrophysiology and Heart Modeling Institute, Fondation Bordeaux Université, Pessac-, Bordeaux, France; 4 Center for Cardiovascular Innovation, University of British Columbia, Vancouver, Canada; 5 Montreal Heart Institute, Université de Montréal, Québec, Canada; 6 Division of Cardiology, University Hospital Southampton NHS Foundation Hospital Trust, Southampton, UK; 7 Unidad de Arritmias, Área de Enfermedades Cardiovasculares, Hospital Universitari i Politècnic La Fe Valencia, and Centro de Investigaciones Biomédicas en RED en Enfermedades Cardiovasculares, Madrid, Spain; 8 Virginia Commonwealth University/Pauley Heart Center, Richmond, Virginia, USA; 9 Department of Medicine, Cardiovascular Disease Division, University of Puerto Rico, San Juan, Puerto Rico 00936 USA; 10 Institute of Medical Statistics and Computational Biology (IMSB), University of Cologne, University Hospital Cologne, Cologne, Germany; 11 Medizinische Klinik I, Abteilung für Kardiologie, Universitätsklinikum Gießen, Gießen, Germany; 12 Department of Internal Medicine II, Ulm University Medical Center, Albert-Einstein-Allee, 23 Ulm, Germany; 13 Electrophysiology Bremen, Klinikum Links der Weser, Bremen, Germany; 14 Department of Cardiac Electrophysiology, Essex Cardiothoracic Centre, Basildon, UK; 15 Circulatory Health Research Group, Medical Technology Research Centre, School of Medicine, Anglia Ruskin University, Chelmsford, CM1 1SQ UK; 16 Department of Cardiology, Maastricht University Medical Center and Cardiovascular Research Institute Maastricht, Maastricht, The Netherlands; 17 Department of Biomedical Sciences, Faculty of Health and Medical Sciences, University of Copenhagen, Copenhagen, Denmark; 18 Caring Futures Institute, College of Nursing and Health Sciences, Flinders University, Adelaide, Australia; 19 Department of Cardiology, University Heart and Vascular Center Hamburg, University Medical Center Hamburg-Eppendorf, Hamburg, Germany; 20 Department of Cardiology, Bern University Hospital and University of Bern, Bern, Switzerland

**Keywords:** Subcutaneous ICD, Implantable cardioverter-defibrillator, Premature battery depletion, Longevity, Advisory

## Abstract

**Background:**

The subcutaneous ICD established its role in the prevention of sudden cardiac death in recent years. The occurrence of premature battery depletion in a large subset of potentially affected devices has been a cause of concern. The incidence of premature battery depletion has not been studied systematically beyond manufacturer-reported data.

**Methods:**

Retrospective data and the most recent follow-up data on S-ICD devices from fourteen centers in Europe, the US, and Canada was studied. The incidence of generator removal or failure was reported to investigate the incidence of premature S-ICD battery depletion, defined as battery failure within 60 months or less.

**Results:**

Data from 1054 devices was analyzed. Premature battery depletion occurred in 3.5% of potentially affected devices over an observation period of 49 months.

**Conclusions:**

The incidence of premature battery depletion of S-ICD potentially affected by a battery advisory was around 3.5% after 4 years in this study. Premature depletion occurred exclusively in devices under advisory. This is in line with the most recently published reports from the manufacturer.

**Trial registration:**

ClinicalTrials.gov Identifier: NCT04767516.

## Introduction

The subcutaneous implantable cardioverter-defibrillator (S-ICD) is established as an alternative to transvenous implantable cardioverter-defibrillators in the prevention of sudden cardiac death [1, 2]. Recently, studies have demonstrated the efficacy and safety of the S-ICD in primary prevention populations, potentially leading to an expansion of the indication criteria [3, 4].

Given the complexities of cardiovascular implantable devices (CIED), it has become relatively commonplace to encounter complications and subsequent advisories regarding CIED components, with lead failure or battery performance being the most common [5, 6]. While the S-ICD was designed to address the limitations of transvenous leads, these devices remain susceptible to advisories related to battery performance. Premature battery depletion (PBD) or failure of the electronic integrity are among the more common causes [7]. Mechanisms of PBD include low-voltage capacitor failure and lithium cluster disposition, as were recently seen in transvenous ICD [8, 9]. The S-ICD has not been an exception from this, with an initial advisory published by Boston Scientific in August of 2019 describing the occurrence of premature battery depletion (PBD) in a small subset of S-ICD generators (Boston Scientific urgent field action REF.92400926-FA). More recently, a higher incidence of PBD was also observed in S-ICD generators that were not part of the initial advisory, which led to a second advisory warning of potential PBD in a subset of more than 35,000 S-ICD (Boston Scientific urgent field action REF.92400926D) [10]. All affected S-ICD devices were manufactured prior to August of 2018, after which the manufacturing process was adapted and the affected low-voltage capacitor switched out. The manufacturer initially projected the incidence to be 3.7% after 5 years.

We sought to provide large-scale, real-life, and manufacturer-independent data regarding the incidence of PBD after S-ICD implantation through a multicenter study of retrospective device data.

## Methods

### Study population and participating centers

This study was a multicenter effort of fourteen centers in Europe, the US, and Canada. Consecutive patients who received an S-ICD at the participating centers were included in this retrospective analysis. Only patients with the models A209 and A219 were included. Data was collected and managed using REDCap electronic data capture tools hosted at the University Hospital Cologne [11]. The study complies with the Declaration of Helsinki. Ethics committee approval was obtained. This study is registered with Clinicaltrials.gov (identifier NCT02241382).

### Collected data and statistical analysis

All data was collected retrospectively from medical files, local registries, and hospital information systems that had been gathered during routine treatment and follow-up.

The primary endpoint in this registry was a composite of device removal, generator replacement, or generator failure (defined as the device reaching elective replacement indicator (ERI) status in patients in whom the device was not removed or replaced subsequently). Reasons for generator removal, replacement, or failure were collected. Device longevity was calculated in months, from generator insertion to the time of generator removal or failure. Premature battery depletion was defined as the occurrence of battery depletion requiring generator replacement after 60 months or less. This definition was chosen since it was previously used in the published literature, and it is in line with the manufacturer’s 5-year longevity warranty.

Continuous variables are provided as mean with standard deviation or median with 25th and 75th percentiles where appropriate. Dichotomous data are provided as numbers with proportion in percentage.

Statistical analyses were performed using CRAN R version 4.2.1 (R Foundation for Statistical Computing, Vienna, Austria) and IBM SPSS Statistics version 26 (Armonk, NY, USA; IBM Corp.).

## Results

The study assessed a total of 1054 S-ICD devices implanted between April 2015 and April 2021. Of these, 597 devices (56.6%) were identified via the Boston Scientific serial number lookup tool (https://www.bostonscientific.com/en-US/pprc/device-lookup-tool.html) as being equipped with a low-voltage capacitor potentially affected by the occurrence of PBD. The mean and median follow-up duration was 2.56 ± 1.62 and 2.4 (IQR: 1.1–3.8) years, respectively. Baseline data is provided in Table 1.

**Table 1:** Baseline characteristics

Cohort [*n*] (%)	1054 (100)
Age [years] (SD)	46.9 ± 15.8
Generator model	
A209 [*n*] (%)	460 (43.6)
A219 [*n*] (%)	594 (56.4)
S-ICD battery	
Under advisory [*n*] (%)	611 (55.4)
Not under advisory [*n*] (%)	491 (44.6)
Indication for ICD therapy	
Primary prevention [*n*] (%)	593 (56.3)
Secondary prevention [*n*] (%)	455 (43.2)
Indication unknown [*n*] (%)	6 (0.5)
Follow-up duration [years] (SD)	2.43 (1.66)

### Device longevity and premature battery depletion

**Fig. 1:**
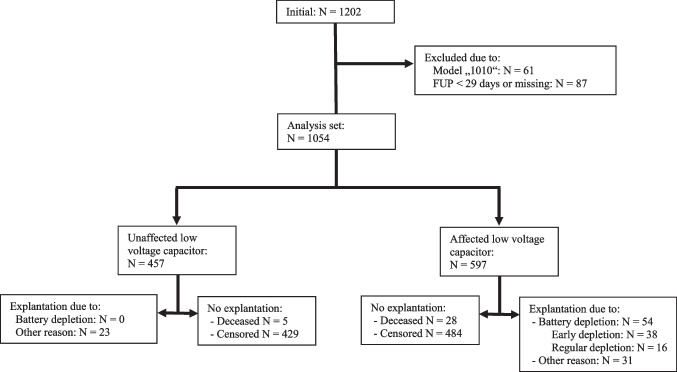
Flowchart of the patients included in the analysis. Legend: FUP, follow-up period

**Fig. 2:**
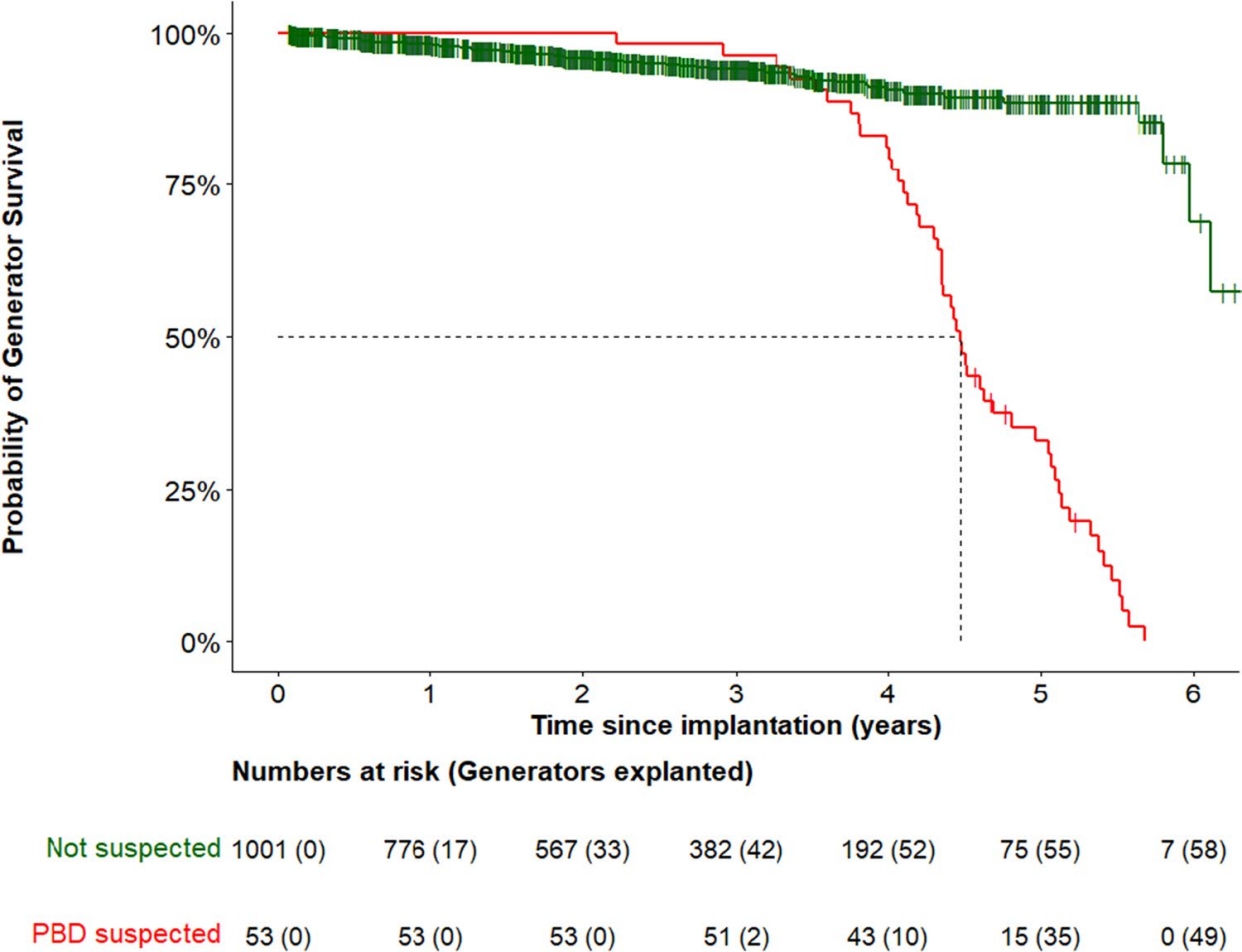
Kaplan-Meier plot of time until generator removal by suspicion of capacitor defect-induced battery failure. Legend: Clinical suspicion of PBD was based on a sudden reduction of battery capacity as reported by the device interrogation

Device failure, replacement, or removal occurred in 108 (10%) cases after a mean time of 3.2 (± 1.7) years. Fifty-four cases (48%) of battery depletion were observed after a mean of 54 (± 9.4) months (median 53 months [48–61]). Regular battery depletion, defined as > 60 months after insertion, occurred in 16 devices (14.2%) after a mean of 64.9 ± 3.6 months. PBD occurred in the remaining 38 (3.5%), where battery failure was seen after a mean of 49 (± 6.8) months. In S-ICD generators not equipped with a potentially faulty capacitor (i.e., not under advisory), no cases of PBD were observed. A flowchart is provided in Fig. 1. A Kaplan-Meier survival curve for the complete cohort is provided in Fig. 2.

### Results of manufacturer’s analysis of explanted devices

The S-ICD generator was sent back to the manufacturer for detailed analysis in 56 cases by the explanting center. Of these, 54 (96%) were under the battery advisory. At the time of data analysis of our registry, the device analysis had been completed by the manufacturer, and the results were provided in 38 cases (86%). In 34 cases, the suspicion of PBD caused by low-voltage capacitor failure was confirmed. Out of these analysis-confirmed cases, in 25 devices the depletion had occurred before the 60 months mark and in 9 devices the depletion had occurred after the 60 months mark, at 5.3 ± 0.2 years. In the remaining 4 analyzed devices, the device had reached ERI shortly after the 5-year mark and the analysis determined regular battery depletion as the cause.

The results of the analysis remained pending in the rest of cases.

### Other causes of device replacement or removal

**Fig. 3:**
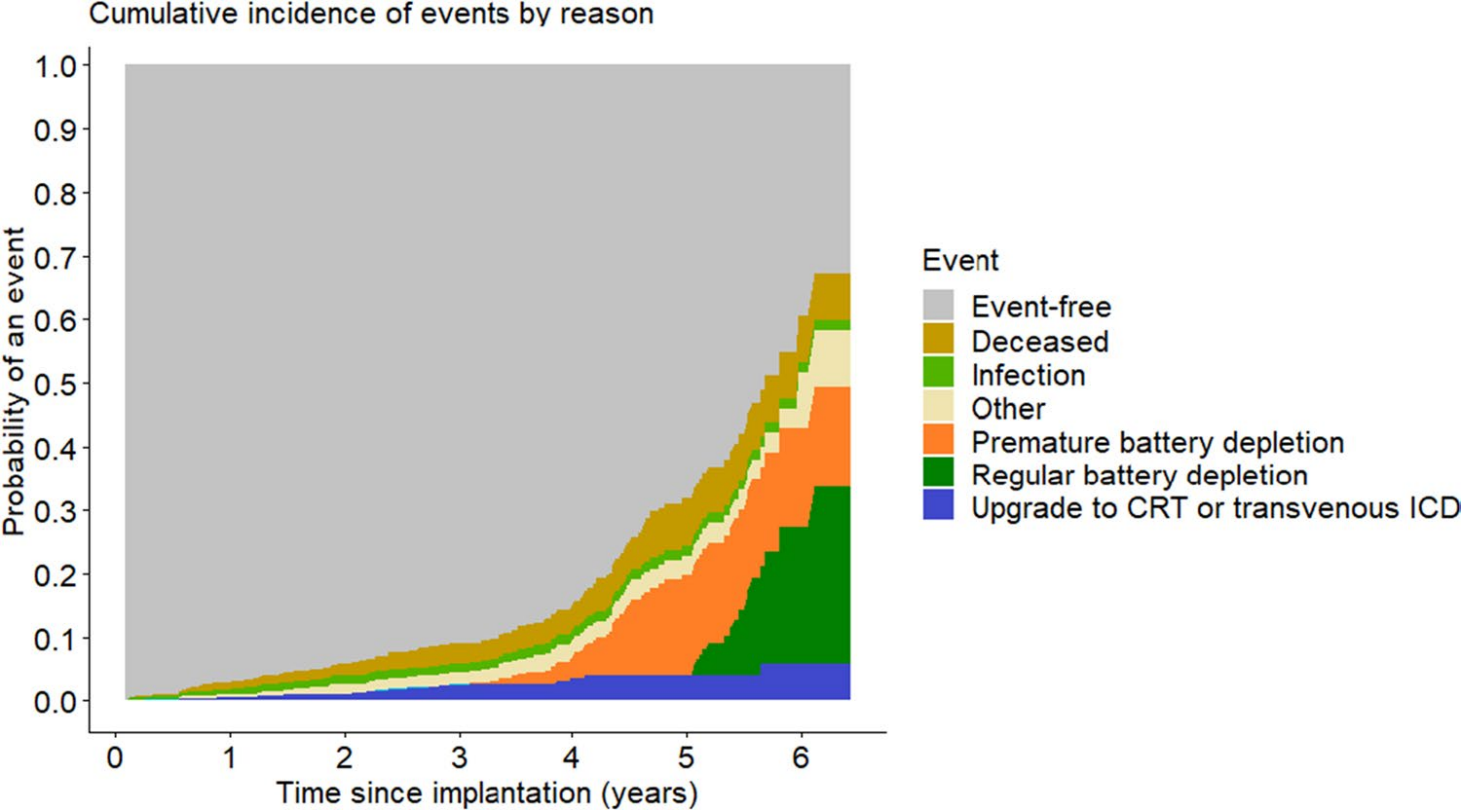
Cumulative events plot of time until generator removal by explantation due to battery depletion vs. due to other reason vs. death before explantation. Legend: CRT, cardiac resynchronization therapy; ICD, implantable cardioverter-defibrillator. “Other” includes heart transplant surgery, left-ventricular assist device surgery, and lead noise or inadequate shocks

In 54 cases (50%), the S-ICD was explanted for reasons other than regular or PBD. Infection (13; 12%) and system upgrade (19; 18%) were the most common indication. Further data regarding these cases is provided in Table 2 and Fig. 3.

**Table 2:** Follow-up data

Duration of follow-up [years] (SD)	2.56 (1.62)
S-ICD explanted during follow-up [*n*] (%)	108 (10)
Indication for S-ICD removal	
Battery depletion	54 (50.0)
- capacitor induced depletion suspected	48 (44.4)
Upgrade to transvenous ICD	19 (17.6)
Infection	13 (12.0)
Upgrade to CRT	3 (2.8)
Heart transplant	5 (4.6)
Inappropriate shocks	4 (3.7)
Inappropriate sensing or noise	3 (2.8)
LVAD therapy	2 (1.9)
Lead failure suspected	2 (1.9)
Not longer indicated	2 (1.9)
Patient discomfort	1 (0.9)
Header failure suspected	1 (0.9)
Unknown	2 (1.9)
Premature battery depletion suspected	53 (5.0)
Removal	49 (92.5)
No removal (patient choice)	4 (7.5)
Devices sent for manufacturer analysis** [*n*] (%)**	56 (53.3)
Low-voltage capacitor failure confirmed	34 (60.7)
No low-voltage capacitor failure	4 (7.1)
Result missing or pending	18 (32.1)

## Discussion

This registry provides the first systematic and manufacturer-independent analysis focusing on the incidence of PBD, defined as battery failure within 60 months, in S-ICD patients. It is the first report of S-ICD registry data with detailed information on S-ICD models affected by the occurrence of PBD in the published literature. Battery depletion occurred in the advisory cohort in 54 cases after a mean of 54 months. PBD defined as 5 years longevity or less occurred in 3.5% of patients after a mean follow-up of 49 (± 6.8) months All devices experiencing battery depletion earlier than 60 months after generator insertion were equipped with a low-voltage capacitor prone to cause PBD, as described in the initial advisory. In 9 devices with a longevity of 5.3 ± 0.2 years that did not meet the PBD endpoint, device analysis confirmed battery depletion due to low-voltage capacitor failure. Out of 34 cases with analysis-confirmed PBD due to low-voltage capacitor failure, 25 (74%) occurred before the 60 months mark and 9 (26%) occurred after

The incidence of PBD in our data is in line with the initial advisory, but seems lower recently updated advisory published by Boston Scientific, which now states the estimated occurrence of PBD at 11.6% after 5 years [12]. This may be explained by the shorter follow-up period of our reported registry data.

No cases of PBD were noted in those devices unaffected by the advisory, as identified with the serial number lookup tool.

### Longevity and incidence of PBD in S-ICD in the literature

The expected longevity of the S-ICD’s battery is 5 to 6 years, as supported by real-world data [13, 14].

Data on the occurrence of PBD in S-ICD patients are sparse. To best of our knowledge, Ip was first to report 4 cases of PBD in a cohort of 118 S-ICD patients [10]. Recently, data from the ELISIR registry was published, reporting PBD incidence of 2.2% over a median of 3.6 years. However, the authors do not provide information what proportion of devices in their cohort was affected by the battery advisory [15].

### Cause and management of premature battery depletion in the S-ICD

The occurrence of PBD in the affected S-ICD generators is caused by latent hydrogen release, leading to a compromise of the function of a low-voltage capacitor. This in turn causes the accelerated depletion of the generator’s battery. When the device reaches the ERI, it is still able to provide therapy delivery for at least 21 days [16].

Unfortunately, with interrogation intervals of 6 months, this may leave patients unprotected due to battery depletion occurring in between visits. The manufacturer’s recommendation was to shorten the interrogation intervals and include all patients in a remote-monitoring database to detect unusual battery behavior and schedule elective replacement. The device’s beeping alert, which will occur after the devices reach the ERI state, should be demonstrated to patients. Of note, this beeping function may be lost after a MRI scan and should thus be tested afterwards. Whether a device is potentially affected can be identified through an online tool provided by the manufacturer (www.BostonScientific.com/lookup).

### Generator removal for reasons other than battery depletion

During follow-up, 54 (5.1%) S-ICD were removed after a mean of 3.18 years. The rate of infection requiring device removal was 1.2%; sensing problems, including noise and inappropriate shocks, were the reason for removal in 0.7%, upgrade to cardiac resynchronization therapy (CRT) or conversion to a transvenous ICD system occurred in 0.3% and 1.8% respectively. These results are comparable with data from the EFFORTLESS registry (2.4% over 3 years) and the ELISIR registry (2% infection after 23 months) [15, 17]. The rate of S-ICD generator removal in our cohort was markedly lower than the 12.9% rate of complete S-ICD system removal after 20 months reported recently by Pothineni and colleagues [18].

## Limitations

Several shortcomings need to be addressed. This was a retrospective multicenter analysis based on data collected during routine patient care and follow-up. Some patients had to be excluded due to missing follow-up data, and the reasons for loss-to-follow-up were unknown. For the deceased patients, no post-mortem device interrogation data was available.

We chose to define PBD in accordance with previous reports and the manufacturer’s longevity warranty as 60 months or less. However, battery depletion due to hydrogen release was confirmed in nine devices with battery depletion shortly after the 60-month mark. Out of the devices with manufacturer analysis-confirmed battery depletion due to low-voltage capacitor failure, this represents 26% of confirmed cases. The ambiguity of the term PBD and the underlying definition needs to be kept in mind when comparing our data with other reports or assessing the performance of the device.

Additionally, our follow-up duration is comparably short to report our findings in a timely fashion. The incidence of depletion is expected to increase towards the 5-year mark; this may explain the significantly higher estimation for the 5-year incidence given by the lasted advisory from Boston Scientific. The focus of this registry was to investigate the occurrence of PBD in the S-ICD population. While data on the reasons for S-ICD generator removal was collected, conclusions towards the incidence of device infection or other reasons for device removal are beyond the scope of this study.

## Conclusion

In this multicenter analysis of retrospective routine follow-up data of S-ICD patients affected by a battery advisory, the incidence of PBD was 3.5% after a mean time of 49 months. This supports the manufacturer’s recently published update of the initial advisory. The manufacturer’s online lookup tool was able to identify all potentially affected devices and no other devices suffered from PBD.

## Abbreviations

ERI: Elective replacement indicator
CIED: Cardiovascular implantable devices
CRT: Cardiac resynchronization therapy
PBD: Premature battery depletion
S-ICD: Subcutaneous implantable cardioverter-defibrillator
